# Sociodemographic, Health and Lifestyle, Sampling, and Mental Health Determinants of 24-Hour Motor Activity Patterns: Observational Study

**DOI:** 10.2196/20700

**Published:** 2021-02-17

**Authors:** Sonia Difrancesco, Harriëtte Riese, Kathleen R Merikangas, Haochang Shou, Vadim Zipunnikov, Niki Antypa, Albert M van Hemert, Robert A Schoevers, Brenda W J H Penninx, Femke Lamers

**Affiliations:** 1 Amsterdam Public Health Research Institute Department of Psychiatry Amsterdam UMC, Vrije Universiteit Amsterdam Netherlands; 2 Interdisciplinary Center Psychopathology and Emotion Regulation Department of Psychiatry, Universitair Medisch Centrum Groningen University of Groningen Groningen Netherlands; 3 Genetic Epidemiology Branch Intramural Research Program National Institute of Mental Health Bethesda, MD United States; 4 Department of Biostatistics, Epidemiology and Informatics University of Pennsylvania Philadelphia, PA United States; 5 Department of Biostatistics Johns Hopkins University Baltimore, MD United States; 6 Department of Clinical Psychology Institute of Psychology Leiden University Leiden Netherlands; 7 Department of Psychiatry Leiden University Medical Center Leiden Netherlands

**Keywords:** actigraphy, functional data analysis, mental health, well-being, activity

## Abstract

**Background:**

Analyzing actigraphy data using standard circadian parametric models and aggregated nonparametric indices may obscure temporal information that may be a hallmark of the circadian impairment in psychiatric disorders. Functional data analysis (FDA) may overcome such limitations by fully exploiting the richness of actigraphy data and revealing important relationships with mental health outcomes. To our knowledge, no studies have extensively used FDA to study the relationship between sociodemographic, health and lifestyle, sampling, and psychiatric clinical characteristics and daily motor activity patterns assessed with actigraphy in a sample of individuals with and without depression/anxiety.

**Objective:**

We aimed to study the association between daily motor activity patterns assessed via actigraphy and (1) sociodemographic, health and lifestyle, and sampling factors, and (2) psychiatric clinical characteristics (ie, presence and severity of depression/anxiety disorders).

**Methods:**

We obtained 14-day continuous actigraphy data from 359 participants from the Netherlands Study of Depression and Anxiety with current (n=93), remitted (n=176), or no (n=90) depression/anxiety diagnosis, based on the criteria of the Diagnostic and Statistical Manual of Mental Disorders, fourth edition. Associations between patterns of daily motor activity, quantified via functional principal component analysis (fPCA), and sociodemographic, health and lifestyle, sampling, and psychiatric clinical characteristics were assessed using generalized estimating equation regressions. For exploratory purposes, function-on-scalar regression (FoSR) was applied to quantify the time-varying association of sociodemographic, health and lifestyle, sampling, and psychiatric clinical characteristics on daily motor activity.

**Results:**

Four components of daily activity patterns captured 77.4% of the variability in the data: overall daily activity level (fPCA1, 34.3% variability), early versus late morning activity (fPCA2, 16.5% variability), biphasic versus monophasic activity (fPCA3, 14.8% variability), and early versus late biphasic activity (fPCA4, 11.8% variability). A low overall daily activity level was associated with a number of sociodemographic, health and lifestyle, and psychopathology variables: older age (*P*<.001), higher education level (*P*=.005), higher BMI (*P*=.009), greater number of chronic diseases (*P*=.02), greater number of cigarettes smoked per day (*P*=.02), current depressive and/or anxiety disorders (*P*=.05), and greater severity of depressive symptoms (*P*<.001). A high overall daily activity level was associated with work/school days (*P*=.02) and summer (reference: winter; *P*=.03). Earlier morning activity was associated with older age (*P*=.02), having a partner (*P*=.009), work/school days (*P*<.001), and autumn and spring (reference: winter; *P*=.02 and *P*<.001, respectively). Monophasic activity was associated with older age (*P*=.005). Biphasic activity was associated with work/school days (*P*<.001) and summer (reference: winter; *P*<.001). Earlier biphasic activity was associated with older age (*P*=.005), work/school days (*P*<.001), and spring and summer (reference: winter; *P*<.001 and *P*=.005, respectively). In FoSR analyses, age, work/school days, and season were the main determinants having a time-varying association with daily motor activity (all *P*<.05).

**Conclusions:**

Features of daily motor activity extracted with fPCA reflect commonly studied factors such as the intensity of daily activity and preference for morningness/eveningness. The presence and severity of depression/anxiety disorders were found to be associated mainly with a lower overall activity pattern but not with the time of the activity. Age, work/school days, and season were the variables most strongly associated with patterns and time of activity, and thus future epidemiological studies on motor activity in depression/anxiety should take these variables into account.

## Introduction

The near ubiquitous use of accelerometers in electronic devices ranging from smartphones to wrist-worn wearables provides the biomedical community with a potential richness of data that is useful for the study of health outcomes. Wrist-worn accelerometers have been used for more than 20 years by sleep researchers to estimate sleep and circadian activity rhythms [1,2], as well as by those studying patterns of physical activity [3]. Research indicates that disruptions in circadian activity rhythms, especially daily motor activity patterns, correlate with poor mental [4] and physical [5] health. Burton et al [6] and our recent results showed that a low level of daily motor activity is associated with depressive [7,8] and anxiety [7] disorders. In addition, sociodemographic and lifestyle factors, especially age and BMI, have been linked to disruptions in daily motor activity patterns. Compared with younger persons, older persons have been found to have lower motor activity patterns [9] and earlier bed and rise times, also known as early chronotype [10]. A higher BMI has been associated with a lower daytime activity level and a higher night-time activity level [11,12]. Circadian activity rhythms are also controlled externally by environmental and social cues. For instance, light is an important synchronizer for circadian activity rhythms [13] and has been shown to be effective in the treatment of sleep disorders [14] and affective disorders [15].

Despite the great interest in daily motor activity patterns and their association with health outcomes and other health factors, commonly used methodologies to analyze actigraphy data are limited in the description of circadian rhythms. Often used validated methods aggregate data in daily indices [16], which loses important information that may be a hallmark of circadian impairment. The traditional approach to actigraphy data analysis employs cosinor (ie, based on the mathematical formula of a cosine wave) or modified cosinor analyses that yield information concerning the amplitude of activity, the timing of “peak” activity, and the goodness-of-fit (ie, how close the pattern is to a cosine wave) [16]. Although they are often quite useful in people with robust activity patterns, these analyses assume the presence of a particular shape of activity (ie, a predictable pattern, such as a cosine waveform) that may be different in individuals with physical or psychological impairments. Functional data analysis (FDA) can be used to model the complete time series of actigraphy data with less restrictive assumptions [17]. Recent studies using functional principal component analysis (fPCA), an FDA technique, have shown that patterns of daily motor activity with specific shapes are associated with psychiatric clinical characteristics (ie, apathy [18], depressive and anxiety symptoms [19], and objectively assessed sleep [19]) in individuals with Alzheimer disease. Another FDA technique that is increasingly being used is function-on-scalar regression (FoSR), which analyzes the relative time-varying associations between each variable of interest and the activity patterns. In addition, this method yields valuable information about the time intervals in which the variables of interest have the greatest influence on activity patterns [20,21]. Banihashemi et al [9] have suggested that older age and higher BMI are linked to lower daytime activity levels and higher BMI and greater symptom severity are associated with nocturnal activity patterns suggestive of sleep disturbances in a population with affective disorders. Those findings are based on first attempts using FDA, and such approaches have not yet been explored in a population with depressive and anxiety disorders. In addition, no studies have extensively assessed the association between actigraphy functional curves and sociodemographic, lifestyle, and sampling factors (eg, season and work/school days). FDA may better capture the complexity and dynamics of daily motor activity to reveal important behavioral biomarkers. This may help us to understand whether intervening on circadian rhythms or sleep (eg, by light therapy or sleep intervention) could be a useful regimen to reduce depressive and anxiety disorders.

In this study, we examined the association between daily activity patterns, assessed using actigraphy and FDA, and (1) sociodemographic (ie, age, sex, partner status, and education level), health and lifestyle (ie, drinking, smoking, chronic diseases, and BMI), and sampling (ie, season, and work/school day versus nonwork/nonschool day) factors, and (2) psychiatric clinical characteristics (ie, current/remitted depressive and anxiety disorders, severity of depressive, and medication use).

## Methods

### Sample

Participants from the Netherlands Study of Depression and Anxiety (NESDA) were selected to enroll in the Ecological Momentary Assessment (EMA) and Actigraphy substudy (NESDA-EMAA). NESDA is one of the cores sites of the Motor Activity Research Consortium for Health (mMARCH) [22,23], a collaborative network for the application of objective assessment of motor activity, sleep, and mood in population and clinical samples. Details about NESDA have previously been discussed extensively [24]. NESDA was designed to investigate the course of depressive and anxiety disorders over a period of several years and the factors that influence the development and prognosis of such disorders. NESDA participants were initially recruited for baseline assessment between 2004 and 2007 (n=2981) and seen for the fifth time at the 9-year follow-up assessment (2014-2017; n=2069) for a regular follow-up interview, including a psychiatric diagnostic interview. In total, 1776 individuals participated in face-to-face interviews. A total of 367 siblings of NESDA participants who met diagnostic criteria for a depressive or anxiety disorder and had the same biological parents as their sibling(s) were included as participants to NESDA’s 9-year follow-up assessment. At the 9-year follow-up, we conducted the EMAA substudy among 384 participants. The NESDA study, including the EMAA component, was approved by the VUmc ethical committee (reference number 2003/183), and all participants gave informed consent for both the regular interview and the EMAA component. A flowchart of the NESDA-EMAA study was previously provided in Difrancesco et al [7]. Eligibility criteria included the following: (1) had a smartphone or were willing to use a smartphone provided by the study, (2) were willing to wear a wrist-worn actigraphy device, and (3) could be enrolled within 1 month of the NESDA interview. Siblings were invited if they did not have a current or past diagnosis of a depressive and/or anxiety disorder or another severe psychiatric disorder (such as psychotic or severe addiction disorder). Participants of the EMAA substudy were provided with a wrist-worn GENEActiv device (Activinsights Ltd) and wore it for 2 weeks on their nondominant wrist. The devices were initialized and set to collect raw activity measures at a frequency of 30 Hz. They also completed questions on their current mood states using EMA [25]. In this paper, we only report on the actigraphy component of the study. Of the 384 participants included in the NESDA-EMAA study, 14 had no available actigraphy data for several reasons, such as technical failure (see [7] for more details), resulting in 370 (96.4%) participants with available data. According to previously published criteria [26], participants’ actigraphy data were included in analyses if at least 1 weekday and 1 weekend day of usable data were available, with at least 16 hours recorded per day and per night. The final sample consisted of 359 (359/384, 93.5%) participants with 13.68 (SD 1.26) valid days; 90.0% (323/359) of participants completed the protocol for 14 days.

### Assessment of Sociodemographic, Health and Lifestyle, and Sampling Factors

Sociodemographic and health and lifestyle factors were assessed at the 9-year follow-up. Sociodemographic factors included age, gender, education level (expressed in years), and partner status. Health and lifestyle factors included BMI (kg/m^2^), number of self-reported chronic diseases under treatment (eg, heart disease, diabetes, stroke, lung disease, osteoarthritis, cancer, ulcer, intestinal problems, liver disease, epilepsy, and thyroid gland disease), number of cigarettes smoked per day, and number of alcoholic drinks consumed per day. Sampling factors were assessed at the 9-year follow-up based on EMA and actigraphy assessment. Sampling factors included whether the actigraphy assessment was performed on a work/school day and the season in which the assessment was performed. Work/school day was identified with information from the EMA assessment. Season was determined based on the date of the actigraphy assessment (eg, 25/09/yyyy=autumn), and winter was used as reference.

### Assessment of Depressive and/or Anxiety Disorders and Clinical Characteristics

As in the previous assessment periods, specially trained clinical research staff conducted the diagnostic interviews at the 9-year follow-up. The Composite International Diagnostic Interview (CIDI; version 2.1) [27] was used to establish diagnoses of depressive disorders (dysthymia and major depressive disorder) and anxiety (social anxiety disorder, panic disorder with and without agoraphobia, agoraphobia, and generalized anxiety disorder) based on the Diagnostic and Statistical Manual of Mental Disorders, fourth edition. For this study, we divided participants into three groups: (1) no lifetime depressive and/or anxiety disorders, (2) remitted depressive and/or anxiety disorders, defined as having a lifetime but not current (6-month) diagnosis, and (3) current depressive or anxiety disorder diagnosed in the past 6 months.

Clinical characteristics that were examined were severity of depressive symptoms and medication use (ie, antidepressant and benzodiazepine use). Severity of depressive symptoms was assessed with the 30-item Inventory of Depressive Symptomatology (IDS) [28]. Antidepressant and benzodiazepine use was based on drug container inspection, and medications were coded according to the Anatomical Therapeutic Chemical (ATC) classification of the World Health Organization. Antidepressant and/or benzodiazepine use was considered present if participants reported using them more than 50% of the time. Antidepressants included selective serotonin reuptake inhibitors (ATC code N06AB), tricyclic antidepressants (ATC code N06AA), and other antidepressants (ATC codes N06AF, N06AG, and N06AX); benzodiazepines included ATC codes N03AE, N05BA, N05CD, and N05CF.

### Statistical Analyses

#### Descriptive Analyses

Distributions of all variables were checked on normality with Q-Q plots. For descriptive statistics, participants’ sociodemographic, health and lifestyle, and sampling factors and clinical characteristics were compared between the three groups (ie, no, remitted, and current depressive and/or anxiety disorders). For normally distributed continuous data, analysis of variance tests were used, and for data with nonnormal distributions, Kruskal-Wallis tests were used. Chi-square tests were used to test differences in frequencies across the three groups. All analyses were performed with the statistical software R (version 1.0.143), and a *P* value of <.05 was considered statistically significant.

#### Assessment of Circadian Rhythm Patterns With fPCA

Raw actigraphy data were processed with open source R package GGIR (version 1.5-18) [29] according to published methods [30,31]. Processing of data included autocalibration, nonwear detection, identification of potentially corrupted data, collapsing of raw data to epoch level, and computation of missing data. Collapsing of raw data to epoch level was done by averaging 5-second data. Minute-to-minute daily actigraphy data were derived per participant by summing these 5-second data; day was defined as the 24-hour clock time interval. As days with at least 16 valid hours were included in the analyses, missing data points were replaced with the participant’s data from the same time of day, averaged across the other valid days, to provide a person-specific informed approach.

Daily motor activity patterns were derived from minute-level actigraphy data with the R package fda for FDA (version 2.4.8) [32]. First, participants’ minute-to-minute actigraphy data was pre-smoothed as linear combinations of a set of nine Fourier basis functions to capture the major trends in daily motor activity (ie, the same procedure as was applied by Zeitzer et al [19] and Gershon et al [33]). Second, fPCA was used to capture the principal directions of daily variation and dimension reduction. fPCA summarized the daily-specific features as the coordinates (called principal component scores) of participants’ curves in the basis spanned by the principal components. The first four daily-specific features, referred to as functional principal components in this paper, were considered because together they explained at least 75% of data variability (a similar cutoff was previously used [33]).

The association between the extracted daily functional principal components for each participant and the participant’s sociodemographic, health and lifestyle, and sampling factors and clinical characteristics were tested by using multiple generalized estimating equation (GEE) regressions, with each functional principal component as the outcome. GEE regression was used to account for correlations between repeated days per person. Separate models were run for each clinical characteristic (ie, current/remitted depressive and/or anxiety disorders, IDS score, and antidepressant use) and each model was adjusted for sociodemographic, health and lifestyle, and sampling factors; this was done to avoid collinearity induced by the high correlation between psychiatric clinical characteristics. Multiple testing correction was applied by controlling the false discovery rate [34].

#### Assessment of Time-Varying Associations of the Activity With Sociodemographic, Health and Lifestyle, and Mental Health Determinants With FoSR

Minute-to-minute daily actigraphy data were aggregated over 10 minutes and averaged over the assessment period for each participant (ie, similar procedure as was used by Goldsmith et al [21]). FoSR was used to study the time-varying associations between participants’ sociodemographic, health and lifestyle, and sampling factors and clinical characteristics, with actigraphy data as the outcome (ie, to study the time-varying associations between several factors and activity patterns). For exploratory purposes, FoSR analysis was repeated for each clinical characteristic to account for the high correlation between psychiatric clinical variables. Each model was adjusted for sociodemographic, health and lifestyle, and sampling factors. Data were analyzed with the R script developed by Goldsmith [35].

## Results

The sociodemographic, health and lifestyle, and sampling factors and clinical characteristics of the study participants are shown in Table 1. Of the total sample (N=359), 93 and 176 participants, respectively, had current and remitted depressive and/or anxiety disorders, while 90 participants had no current depressive and/or anxiety disorders. The current depressive/anxiety disorder group was heterogeneous in that 38.7% (36/93) had anxiety disorders only, 33.3% (31/93) had depressive and anxiety disorders, and 28.0% (26/93) had depressive disorders only. As expected, individuals with current depressive and/or anxiety disorders scored significantly higher on depressive symptoms (*P*<.001) and used antidepressants more frequently than individuals in the other groups, although no significant differences were found for benzodiazepine use.

**Table 1:** Participants’ sociodemographics, health and lifestyle factors, actigraphy assessment and psychiatric characteristics, and medication use (N=359).

Variables		Current depressive and/or anxiety disorders (n=93)	Remitted depressive and/or anxiety disorders (n=176)	No depressive and/or anxiety disorders (n=90)	*P* value
**Sociodemographics**					
	Age, mean (SD)^a^	50.1 (11.1)	48.2 (13.4)	51.3 (12.5)	.13
	Female, n (%)^b^	58 (62.4)	120 (68.2)	50 (55.6)	.12
	Education level (years), mean (SD)^a^	12.5 (3.4)	12.7 (2.8)	13.9 (2.9)	<.001
	Has a partner, n (%)^b^	45 (48.4)	90 (51.1)	51 (56.7)	.52
**Lifestyle factors** ^a^					
	BMI, mean (SD)	27.1 (5.1)	26.6 (5.2)	26 (5.4)	.37
	Number of chronic diseases, mean (SD)	1.1 (1.2)	1.0 (1.1)	0.6 (0.8)	.008
	Number of cigarettes per day, mean (SD)	3.2 (6.5)	2.9 (6.1)	0.8 (2.7)	.004
	Number of drinks per day, mean (SD)	0.5 (0.8)	0.7 (1.2)	0.8 (0.8)	.16
**Actigraphy assessment characteristics**					
	Number of actigraphy days, mean (SD)^a^	13.7 (1.0)	13.6 (1.5)	13.8 (0.7)	.48
	Measures on work/school days, n (%)^b^	398 (34.1)	769 (37.2)	467 (42.8)	<.001
**Season of actigraphy measurement** ^b^					<.001
	Winter, n (%)	277 (21.8)	701 (29.3)	369 (29.7)	
	Autumn, n (%)	376 (29.5)	584 (24.4)	362 (29.1)	
	Spring, n (%)	432 (33.9)	619 (25.8)	260 (20.9)	
	Summer, n (%)	188 (14.8)	492 (20.5)	252 (20.3)	
**Psychopathology**					
	Only depressive disorders, n (%)	26 (28.0)	46 (26.1)	N/A^c^	
	Only anxiety disorders, n (%)	36 (38.7)	24 (13.6)	N/A	
	Depressive and anxiety disorders, n (%)	31 (33.3)	106 (60.2)	N/A	
	Inventory of Depressive Symptomatology, mean score (SD)^a^	28.6 (11.4)	20.9 (12.5)	6.0 (4.9)	<.001
**Medication use**					
	Antidepressant users, n (%)^b^	35 (37.2)	34 (19.3)	2 (2.2)	<.001
	Benzodiazepine users, n (%)^b^	5 (5.3)	8 (4.5)	0 (0.0)	.10

^a^Analysis of variance tests were performed.

^b^Chi-square tests were performed.

^c^N/A: not applicable.

**Figure 1:**
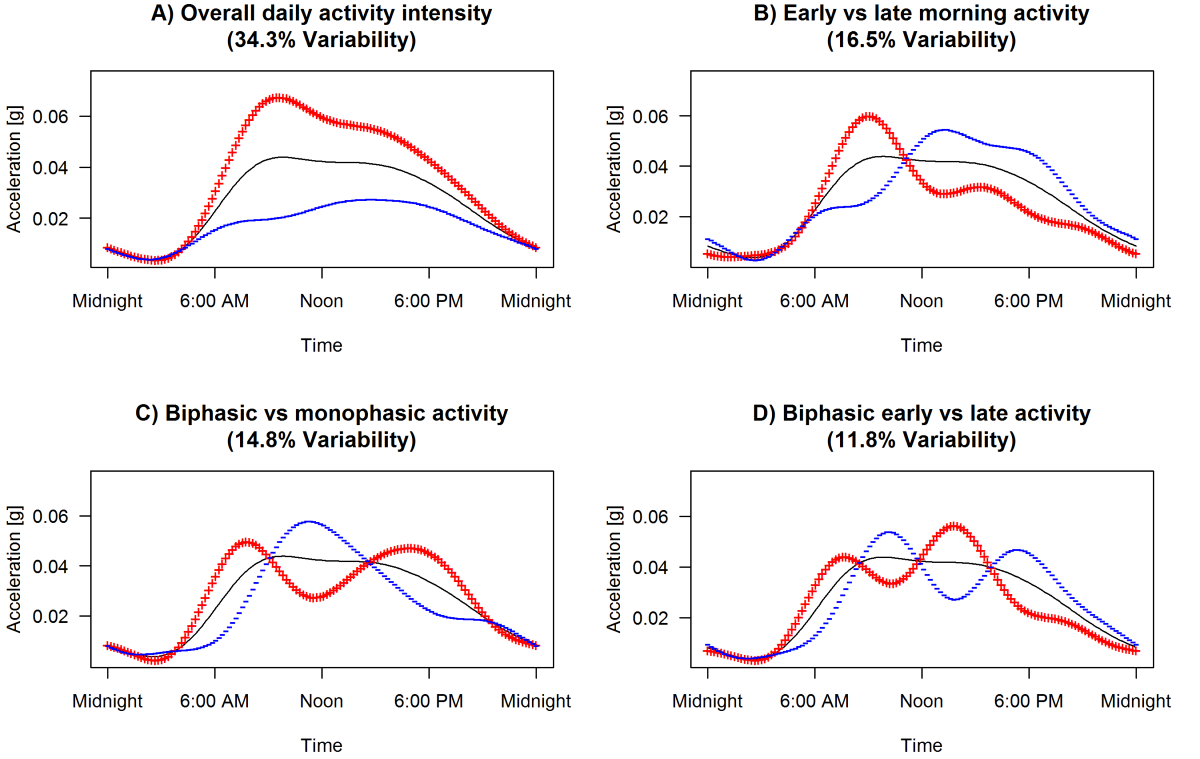
Patterns of daily activity explaining 77.4% of variability in the data (N=359). The black line represents the average daily activity, the red line represents the average daily activity plus 1 SD of the functional principal component analysis (fPCA) score, and the blue line represents the average daily activity minus 1 SD of the fPCA score. (A) High (+) versus low (–) overall daily activity intensity. (B) Early (+) versus late (–) morning activity. (C) Biphasic (+) versus monophasic (–) activity. (D) Early (+) versus late (–) biphasic activity. Determinants associated with a pattern marked with (+) have a positive β value; if not, they have a negative β value.

Four components of daily activity patterns that explained 77.4% of the variability in the data were extracted using fPCA (Figure 1) and interpreted as follows. The first component was the overall daily activity level. The second component described early versus late morning activity and could be indicative of chronotype. The third component showed a biphasic versus monophasic activity pattern, while the fourth component represented an early versus late biphasic activity pattern. The biphasic pattern showed two cycles of increased activity with subsequent decreased activity.

A low overall daily activity level was associated with a number of sociodemographic, health and lifestyle, and psychopathology variables (Table 2): older age (*P*<.001), higher education level (*P*=.005), higher BMI (*P*=.009), greater number of chronic diseases (*P*=.02), greater number of cigarettes smoked per day (*P*=.02), current depressive and/or anxiety disorders (*P*=.05), and greater severity of depressive symptoms (*P*<.001). A high overall daily activity level was associated with work/school days (*P*=.02) and summer (reference: winter; *P*=.03) (Table 2). Earlier morning activity was associated with older age (*P*=.02), having a partner (*P*=.009), work/school days (*P*<.001), and autumn and spring (reference: winter; *P*=.02 and *P*<.001, respectively) (Table 2). Monophasic activity was associated with older age (*P*=.005; Table 2). Biphasic activity was associated with work/school days (*P*<.001) and summer (reference: winter; *P*<.001) (Table 2). Earlier biphasic activity was associated with older age (*P*=.005), work/school days (*P*<.001), and spring and summer (reference: winter; *P*<.001 and *P*=.005, respectively) (Table 2).

**Figure 2:**
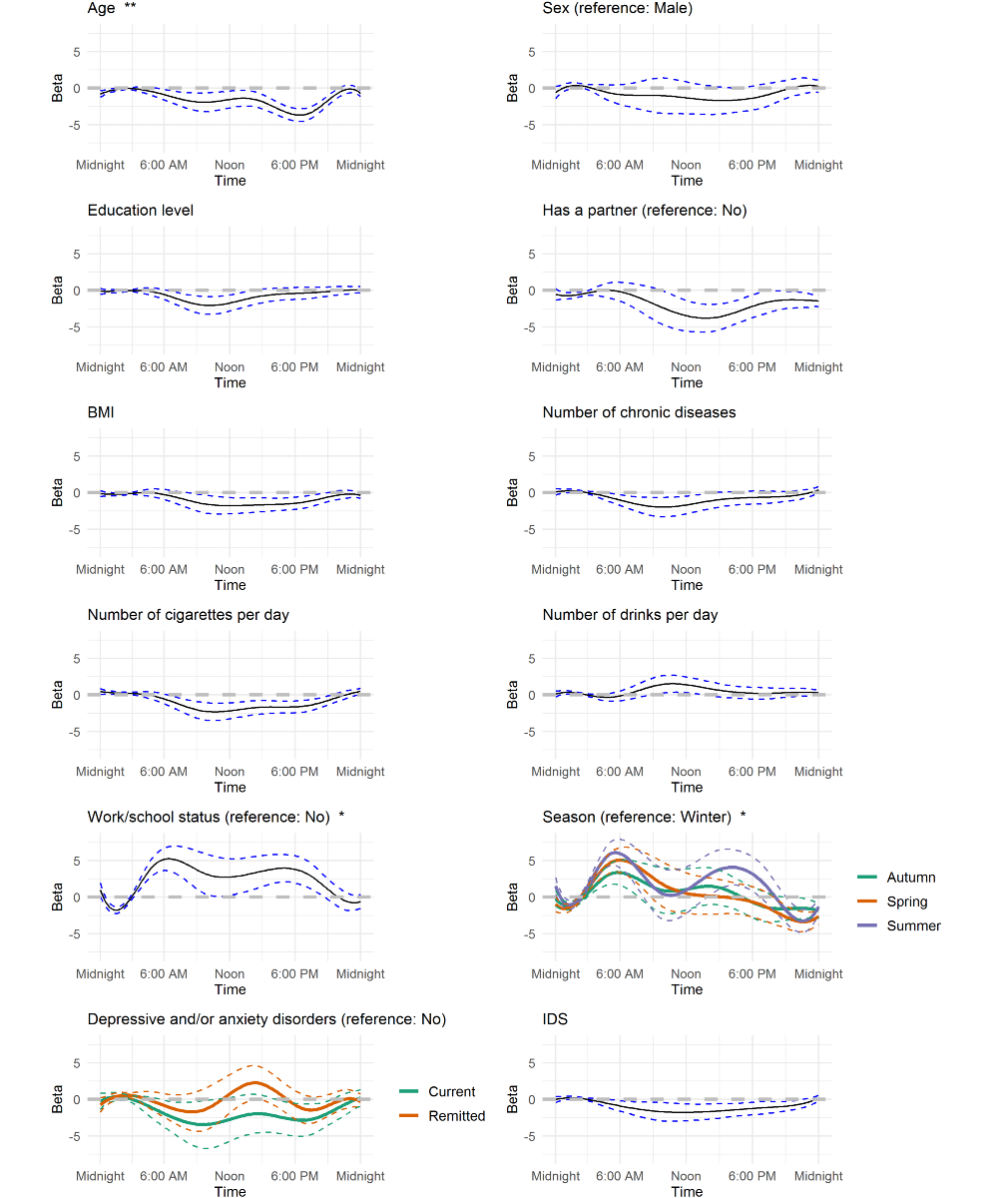
Effect of sociodemographics, health and lifestyle factors, and actigraphy and clinical characteristics on time of activity from multivariable function-on-scalar regression analyses. IDS: Inventory of Depressive Symptomatology. *P<.05; **P<.01.

**Figure 3:**
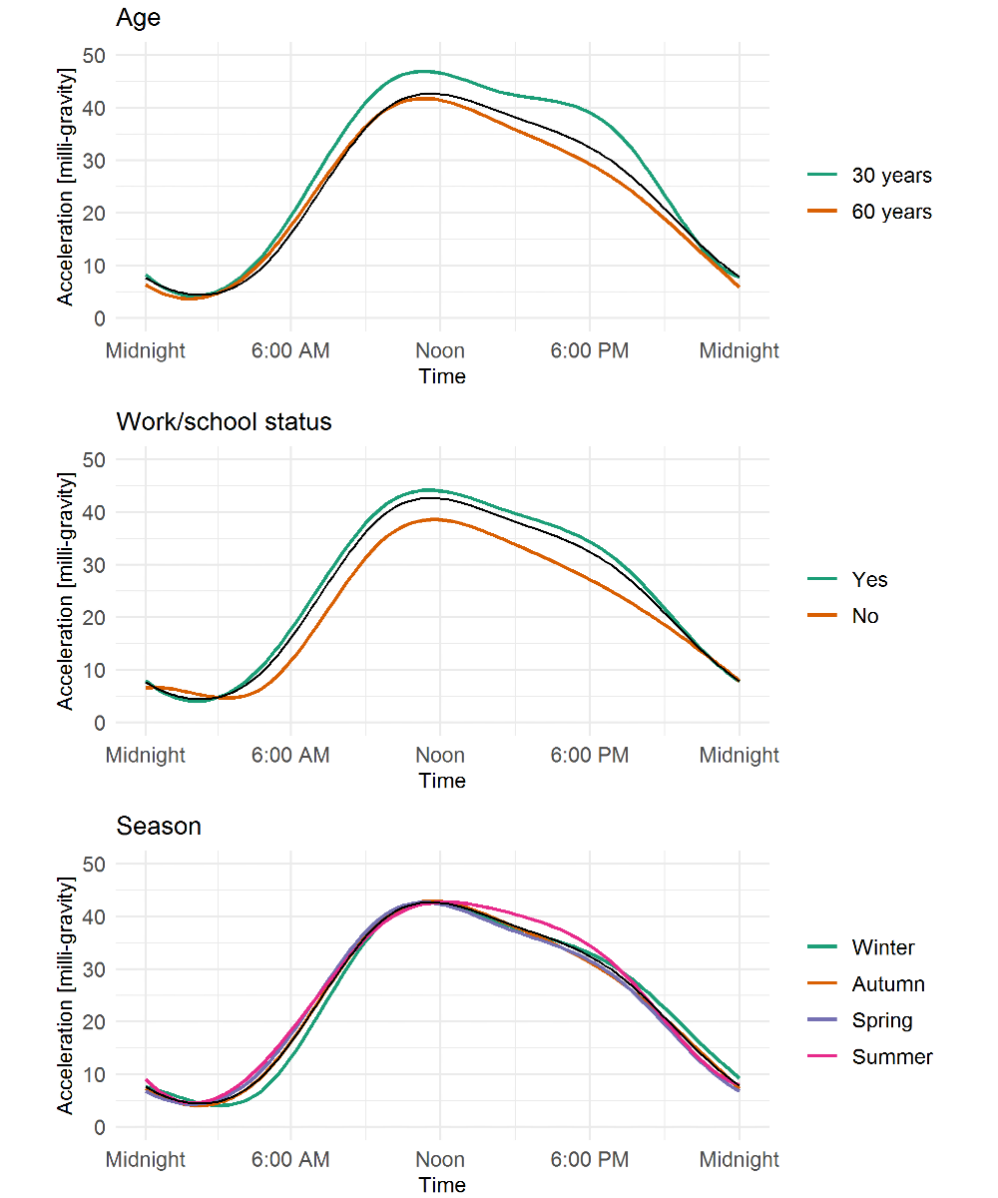
Average daily motor activity curves by age, work/school status, and season.

Age, work/school status, and season were significantly associated with motor activity in the FoSR analyses (Figure 2; *P*<.05). Older age was especially related to lower activity in the late afternoon (around 6 PM). Working or going to school was associated with a higher activity level, with a dip in activity level around noon, compared with not working/going to school (Figure 2). Compared with individuals assessed in winter, those assessed in summer, spring, and autumn had higher activity levels in the morning and those assessed in the summer had higher activity levels late in the afternoon (Figure 2). Average daily motor activity curves by age, work/school status, and season are shown in Figure 3.

**Table 2:** Multivariable associations between daily motor activity patterns and sociodemographics, health and lifestyle factors, actigraphy assessment characteristics, and psychopathology (N=359).^a^

Variables			Overall daily activity intensity			Early vs late morning activity			Biphasic vs monophasic activity				Early vs late biphasic activity			
			β	SE	*P*	β	SE	*P*		β	SE	*P*		Β	SE	*P*
**Sociodemographics**																
	Age		–.15	0.04	<.001	.08	0.03	.02		–.10	0.03	.005		.10	0.03	.005
	**Sex**															
		Female	Ref^b^			Ref				Ref				Ref		
		Male	–.05	0.08	.65	–.02	0.06	.83		–.03	0.06	.79		–.07	0.06	.34
	Education level		–.13	0.04	.005	–.01	0.03	.80		.04	0.03	.30		–.03	0.03	.54
	**Has a partner**															
		No	Ref			Ref				Ref				Ref		
		Yes	–.17	–0.08	.07	.18	–0.06	.009		–.02	–0.05	.83		.05	–0.05	.496
**Lifestyle factors**																
	BMI		–.12	0.04	.009	.05	0.03	.22		–.01	0.02	.83		.01	0.03	.80
	Number of chronic diseases		–.11	0.04	.02	–.01	0.03	.82		–.03	0.03	.47		–.05	0.03	.15
	Number of cigarettes per day		–.14	0.05	.02	.03	0.03	.55		–.05	0.03	.19		.02	0.03	.69
	Number of drinks per day		.08	0.05	.19	–.05	0.04	.40		–.04	0.03	.34		–.01	0.03	.83
**Actigraphy assessment characteristics**																
	**Work/school day**															
		No	Ref			Ref				Ref				Ref		
		Yes	.17	0.06	.02	.28	0.05	<.001		.73	0.05	<.001		.19	0.05	<.001
	**Season**															
		Winter	Ref			Ref				Ref				Ref		
		Autumn	.10	0.09	.39	.19	0.07	.02		.02	0.07	.83		.14	0.06	.07
		Spring	.15	0.09	.21	.33	0.07	<.001		.14	0.07	.08		.26	0.07	<.001
		Summer	.29	0.11	.03	.18	0.10	.19		.33	0.07	<.001		.24	0.08	.005
**Psychopathology**																
	**Depressive and/or anxiety disorders**															
		No	Ref			Ref				Ref				Ref		
		Remitted	–.06	0.08	.60	–.04	0.08	.79		–.05	0.07	.64		.08	0.07	.39
		Current	–.24	0.10	.05	.02	0.08	.83		–.001	0.08	.99		–.01	0.07	.95
		IDS^c^	–.14	0.04	<.001	–.05	0.04	.34		–.02	0.03	.70		–.04	0.03	.30
	**Antidepressant use**															
		No	Ref			Ref				Ref				Ref		
		Yes	–.17	0.11	.22	–.12	0.06	.14		–.08	0.07	.38		–.11	0.08	.33

^a^Multivariable generalized estimating equation models were run and each model was adjusted for sociodemographic, health and lifestyle, and sampling factors. Multiple testing correction was applied using false discovery rate estimation.

^b^Ref: reference.

^c^IDS: Inventory of Depressive Symptomatology.

## Discussion

### Principal Findings

This is the first study to examine the associations between patterns of daily motor activity and sociodemographic, health and lifestyle, and sampling factors and psychiatric clinical characteristics in individuals with and without depressive and anxiety disorders using FDA. Patterns of daily motor activity extracted with fPCA seem to reflect commonly studied circadian activity rhythm features such as daily activity level and time-of-day preference for morningness or eveningness [36]. The presence and severity of depressive and anxiety disorders were associated with a low overall activity pattern but had no impact on time of activity. Sociodemographic, health and lifestyle, and sampling factors were independently associated with daily motor activity patterns. FoSR analyses indicated that age, work/school status, and season of assessment were associated with time of activity.

### Comparison With Previous Literature

The majority of the variability in the data (77.4%) was explained by four principal components that reflect the complexity of activity patterns. In line with previous studies employing fPCA, the first two components were commonly studied actigraphy features related to circadian rhythms: overall level of physical activity [18,19,33] and time-of-day preference for morningness or eveningness [33]. Interpretation of the third and fourth components was less clear. We found a monophasic versus biphasic activity pattern, as previously reported in other studies [19,33], where it was suggested that a biphasic rhythm may reflect napping behavior. This monophasic pattern could also be related to the temporary drop in alertness and performance that often occurs during the early afternoon, referred to as the postlunch dip, which reflects the 12-hour harmonic of the circadian clock [37]. Therefore, the level of daily activity and chronotype seem to be consistent components across studies applying fPCA, demonstrating the generalizability of the extracted components and confirming that these are important features of daily activity patterns.

Sociodemographic factors, especially age, were associated with daily motor activity patterns with a low activity level. This is in line with the results of the study by Takagi et al [38]. This may be due to the age-related decline in physical activity [39]. Aging causes changes in the organism that lead to a gradual loss of function, frailty, disease, and disability [40], and therefore result in decreased physical activity and physical functioning. The sleep-wake cycle also appears to change in the aging process. Our findings showing the association between earlier rhythms and increasing age are consistent with previous research that shows that aging is associated with advanced sleep timing (as reviewed by Duffy et al [41]) and a preference for morningness (as reviewed by Hood et al [42]). The circadian phase of melatonin has also been reported to become earlier with age, as has the timing of the cortisol rhythm [41]. The suprachiasmatic nucleus (SCN), which represents the biological clock of the brain, shows functional changes with age [43] that may be related to disturbances in circadian rhythms.

Work/school status and season, not surprisingly, were also very important to daily activity patterns and circadian rhythms. Circadian rhythms are controlled centrally by the SCN and influenced externally by behavioral/social cues and by light exposure, as reviewed by Duffy and Czeisler [44]. Our finding that assessments performed on autumn, spring, and summer days showed significantly higher overall levels of activity and higher levels of early morning activity than assessments performed on winter days seems to be consistent with the systematic review of Tucker and Gilliland [45], which showed that level of activity varies with seasonality and is the lowest during winter. Also, other factors related to the season can have an impact on daily activity such as weather conditions, which can also explain different circadian patterns across seasons. Also, on work/school days, there appeared to be higher and more sustained levels of daily activity and earlier morning activity compared with on nonwork/nonschool days. Indeed, it is well known in the literature that modern life habits including night work, shift work [46], jet lag [47], and social jet lag [48] are associated with circadian rhythm disruptions.

We also found that health and lifestyle factors are linked to daily activity patterns. Our results are in line with previous research reporting an association between lower activity levels and higher BMIs in the NIMH Family study [12] and greater numbers of chronic diseases [49]. These results may be suggestive of sedentary behavior, a factor known to relate to weight gain and disabilities. Early morning activity was associated with higher BMIs. This might be because of respiration-related diseases such as apnea, which is known to be more prevalent in persons with a high BMI and can severely disturb sleep [50].

An important aspect of this research was to study the association between timing of daily motor activity and psychopathology. However, we found no association with the timing of the activity. Instead, our functional data-driven models showed similar associations as were found in our previous analyses on NESDA data [7,8], indicating that current depressive and/or anxiety disorders and more severe symptoms were associated with lower physical activity levels but not with a preference for eveningness. These results suggest that the use of daily indices of motor activity may be sufficient when studying the association with psychopathology. On the other hand, we have only evaluated group-level differences; studying differences at the individual level may be important to explore. For instance, by using an idiographic approach (ie, study associations that differ between time points or between individuals), it may be possible to study the dynamics between daily motor activity and depression and/or anxiety [51] and help identify patients in whom activity is strongly predictive of mood. Collecting more empirical data in clinical practice is necessary to establish whether this is a promising approach.

### Strengths of the Study

An important strength of this paper is the use of FDA, which is a useful statistical tool for data exploration and visualization. By providing a graphical representation of motor activity and circadian rhythms, FDA can help to identify specific patterns. This could help to generate new hypotheses that could, in turn, contribute to an improvement of the treatment of circadian disturbances. FDA could also be used to predict future outcomes of treatment. For instance, the study of Zeitzer et al [19] showed that low daytime activity and a late afternoon peak extracted with fPCA are predictive of higher mortality rates in community-dwelling older men, although it remains to be investigated whether fPCA components add to the prediction over traditional actigraphy measures. Daily curves of motor activity could potentially also be used in predictive models to pick up early signs of recovery or nonresponse and, if predictive, could inform clinicians in monitoring treatment response or treatment planning.

### Limitations

This study was limited by several factors. The data were observational and cross-sectional, and thus the associations cannot be inferred to be causal. As the subjects in this study were individuals participating in the fifth wave of a prospective cohort, there may be a selection bias toward highly motivated individuals. Future studies may investigate whether our results may be replicated in a wider population with and without depression and anxiety. Although actigraphy provides only an indirect assessment of circadian rhythm, it has the advantage of continuously monitoring over a relatively long period of time. Not all functional components were easily interpretable. While the first two components are (possibly) indicative of the overall level of activity and the time-of-day preference for morningness or eveningness, the third and fourth components need to be replicated in order to provide a better interpretation and greater validity.

### Conclusions

Our study showed that features of daily motor activity extracted with fPCA reflect commonly studied factors such as the daily activity level and the time-of-day preference for morningness or eveningness. Age, work/school status, and season were the variables most strongly associated with patterns of daily activity and had time-varying associations with daily motor activity. The presence and severity of depressive and anxiety disorders were associated with a lower overall activity level but not with the timing of activity. Other than psychopathology, sociodemographic, health and lifestyle, and sampling factors were independently associated with a low overall activity pattern.

## Abbreviations

ATC: Anatomical Therapeutic Chemical
CIDI: Composite International Diagnostic Interview
EMA: ecological momentary assessment
FDA: functional data analysis
FoSR: function-on-scalar regression
fPCA: functional principal component analysis
GEE: generalized estimating equation
IDS: Inventory of Depressive Symptomatology
mMARCH: Motor Activity Research Consortium for Health
NESDA: Netherlands Study of Depression and Anxiety
NESDA-EMAA: Ecological Momentary Assessment and Actigraphy substudy of the Netherlands Study of Depression and Anxiety
SCN: suprachiasmatic nucleus
